# Rotenone Modulates *Caenorhabditis elegans* Immunometabolism and Pathogen Susceptibility

**DOI:** 10.3389/fimmu.2022.840272

**Published:** 2022-02-22

**Authors:** Danielle F. Mello, Christina M. Bergemann, Kinsey Fisher, Rojin Chitrakar, Shefali R. Bijwadia, Yang Wang, Alexis Caldwell, Larry Ryan Baugh, Joel N. Meyer

**Affiliations:** ^1^ Nicholas School of the Environment, Duke University, Durham, NC, United States; ^2^ Department of Biology, Duke University, Durham, NC, United States; ^3^ Center for Genomic and Computational Biology, Duke University, Durham, NC, United States

**Keywords:** immunotoxicity, metabolism, innate immunity, mitoimmunity, invertebrate, mitochondrial toxicants, pesticides

## Abstract

Mitochondria are central players in host immunometabolism as they function not only as metabolic hubs but also as signaling platforms regulating innate immunity. Environmental exposures to mitochondrial toxicants occur widely and are increasingly frequent. Exposures to these mitotoxicants may pose a serious threat to organismal health and the onset of diseases by disrupting immunometabolic pathways. In this study, we investigated whether the Complex I inhibitor rotenone could alter *C. elegans* immunometabolism and disease susceptibility. *C. elegans* embryos were exposed to rotenone (0.5 µM) or DMSO (0.125%) until they reached the L4 larval stage. Inhibition of mitochondrial respiration by rotenone and disruption of mitochondrial metabolism were evidenced by rotenone-induced detrimental effects on mitochondrial efficiency and nematode growth and development. Next, through transcriptomic analysis, we investigated if this specific but mild mitochondrial stress that we detected would lead to the modulation of immunometabolic pathways. We found 179 differentially expressed genes (DEG), which were mostly involved in detoxification, energy metabolism, and pathogen defense. Interestingly, among the down-regulated DEG, most of the known genes were involved in immune defense, and most of these were identified as commonly upregulated during *P. aeruginosa* infection. Furthermore, rotenone increased susceptibility to the pathogen *Pseudomonas aeruginosa* (PA14). However, it increased resistance to *Salmonella enterica* (SL1344). To shed light on potential mechanisms related to these divergent effects on pathogen resistance, we assessed the activation of the mitochondrial unfolded protein response (UPR^mt^), a well-known immunometabolic pathway in *C. elegans* which links mitochondria and immunity and provides resistance to pathogen infection. The UPR^mt^ pathway was activated in rotenone-treated nematodes further exposed for 24 h to the pathogenic bacteria *P. aeruginosa* and *S. enterica* or the common bacterial food source *Escherichia coli* (OP50). However, *P. aeruginosa* alone suppressed UPR^mt^ activation and rotenone treatment rescued its activation only to the level of DMSO-exposed nematodes fed with *E. coli*. Module-weighted annotation bioinformatics analysis was also consistent with UPR^mt^ activation in rotenone-exposed nematodes consistent with the UPR being involved in the increased resistance to *S. enterica*. Together, our results demonstrate that the mitotoxicant rotenone can disrupt *C. elegans* immunometabolism in ways likely protective against some pathogen species but sensitizing against others.

## Introduction

Increasing evidence reveals that different classes of environmental toxicants can have substantial effects on the function and homeostasis of the immune system of multiple animal species [see reviews (1–7)]. The complexity of the immune system provides a multitude of possible mechanisms by which toxicants may impact immune-related responses. Immunotoxicity may occur through direct interactions with molecules implicated in canonic immunological pathways, or by disrupting the crosstalk between the immune system and other physiological systems (2).

Recent research has shown that energy metabolism and immune responses are intimately connected: Metabolic shifts may directly modulate immune function, and molecules and cells of the immune system regulate metabolic pathways (8). These connections are studied by the recently established research field of immunometabolism (9). Studies in immunometabolism have revealed mitochondria as a key modulator of immune responses (10), which can be also defined as mitoimmunity (11). Some of the main findings regarding the mitochondrial control of immune responses reveal that (i) mitochondrial components, such as mitochondrial DNA and cardiolipin, act as a danger-associated molecular pattern (DAMP) activating cell-autonomous and cell non-autonomous immune responses; (ii) molecules present at the mitochondrial outer membrane form a platform for anti-viral signaling and inflammasome activation; (iii) mitochondrial dynamics (biogenesis, fusion, and fission) have roles in immune-cell activation; and (iv) Krebs cycle intermediates are necessary for immune-related responses and inflammation, in both innate and adaptive immune cells (12). Therefore, it is reasonable to consider that toxicant-induced mitochondrial damage or disruption may have profound effects on the homeostasis of the immune system. Indeed, increasing evidence suggests that mitochondrial dysfunction caused by environmental toxicants is implicated in the onset of several diseases and immune-related disorders (13). Moreover, mitochondria can be particularly vulnerable targets to several classes of environmental toxicants, including metals, polycyclic aromatic hydrocarbons (PAHs), and pesticides (14, 15). Despite this awareness, little is known about how mitotoxicants affect immunometabolic processes.

**Figure 1:**
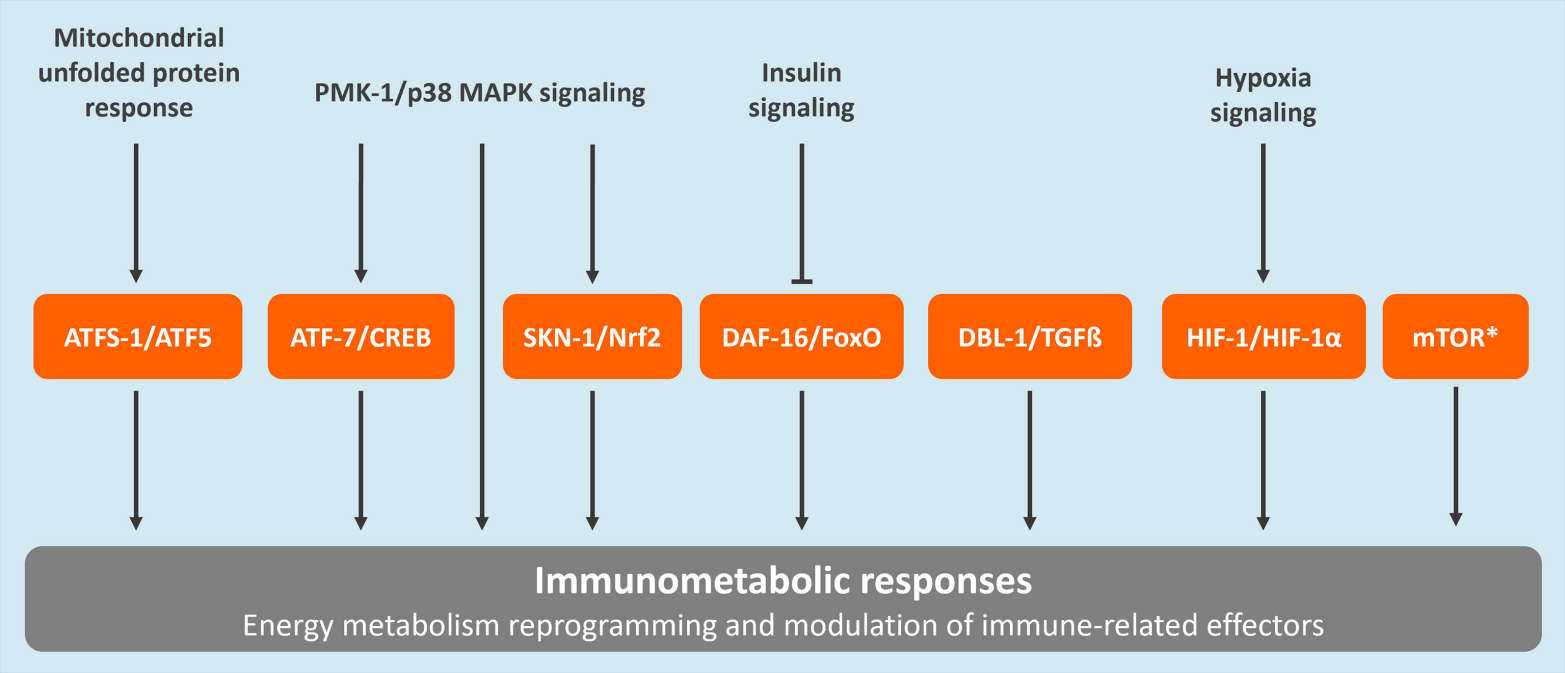
Conserved regulatory pathways associated to immunometabolism in *C. elegans*. These include the mitochondrial unfolded protein response (UPRmt), the p38 mitogen-activated protein kinase (MAPK) signaling pathway, the insulin/insulin-like growth factor signaling (IIS) pathway, the forkhead box transcription factor O (FoxO)-mediated signaling, the transforming growth factor-beta (TGFβ) signaling pathway, the nuclear factor erythroid 2-related factor 2 (Nrf2) pathway, the hypoxia-Inducible factor (HIF)-1 regulatory pathway, and the mammalian target of rapamycin (mTOR) pathway. The *C. elegans* protein names are provided followed by the corresponding human homolog. *The mTOR pathway is regulated by two complexes, TORC1 and TORC2. The main proteins composing these complexes, which are conserved between *C. elegans* and humans, are the following: LET-363/TOR, DAF-15/Raptor, and RICT-1/Rictor (20).

*Caenorhabditis elegans*, a free-living nematode, has traditionally been utilized in the areas of neuroscience, aging, and developmental biology. Over the past few decades, this model has also been recognized as a valuable tool in revealing fundamental principles in innate immunity and host-pathogen interactions (16). Most recently, *C. elegans* was also established as an important model for unraveling key concepts in immunometabolism (17). Most of the success in the use of *C. elegans* in research relies on the fact that this species is a valuable representative of metazoans, including humans. For example, 83% of the worm proteome contains human homologs (18, 19). Importantly, various molecular pathways involved in immunometabolic responses in mammals are conserved in *C. elegans* (**Figure 1**) (17, 20, 21). Moreover, the key role of mitochondria as a signaling hub for innate immune responses also seems to be conserved between vertebrates and *C. elegans* (22, 23).

Over the past years, *C. elegans* has also emerged as a prevalent toxicological test organism, being successfully used in drug discovery screens (24) and shown to be predictive of chemical toxicity in mammals, with several reported cases of a conserved mode of toxic action (24). *C. elegans* has also been proposed as a prominent model in environmental toxicological studies (25, 26). Thus, research using this model may reveal valuable insights regarding immunometabolism toxicity mechanisms and environmental health.

In this study, we exposed nematodes to the pesticide rotenone, a classic complex I inhibitor, to investigate the role of specific mitochondrial disruption on modulating conserved immuno-metabolic molecular pathways and disease susceptibility. We found that rotenone disrupted nematode growth and mitochondrial bioenergetics, as expected, and modulated the expression of genes and pathways mostly involved in detoxification, energy metabolism, and pathogen defense, including genes related to conserved immunometabolic pathways such as UPR^mt^ and HIF. Among the down-regulated DEG, most of the known genes were involved in immune defense, revealing a major role of rotenone in suppressing immune-related genes, which (according to the WormExp gene overlap analysis tool) included genes upregulated by *Pseudomonas aeruginosa* resistance. In line with these results, rotenone-exposed animals were more susceptible to *P. aeruginosa* (PA14) challenges. In contrast, rotenone rendered animals more resistant when challenged with another pathogen, *Salmonella enterica* (SL1344). Finally, by evaluating the expression of the UPR^mt^ reporter gene *hsp-6*, we propose that the UPR^mt^ pathway might mediate rotenone-induced resistance to *S. enterica*.

## Materials and Methods

### *C. elegans* and Bacterial Strains

Bristol N2 and SJ4100 (zcIs13[*hsp-6p::gfp*]) strains, were obtained from the Caenorhabditis Genetics Center (CGC). The following bacterial strains were used in this study: *Escherichia coli* OP50-1 [SmR] and HB101, which were obtained from the CGC, and *P. aeruginosa* (PA14) and *S. enterica* serovar Typhimurium 1344 (SL1344), which were provided by the laboratory of Alejandro Aballay. All *C. elegans* strains used were grown and maintained at 20°C on K-agar plates (51 mM sodium chloride, 32 mM potassium chloride, 1 mM calcium chloride, 1 mM magnesium sulfate, 10 µg/mL cholesterol, 0.25% peptone, 2% agar) seeded with OP50 *E. coli* bacteria.

### Rotenone Exposures

**Figure 2:**
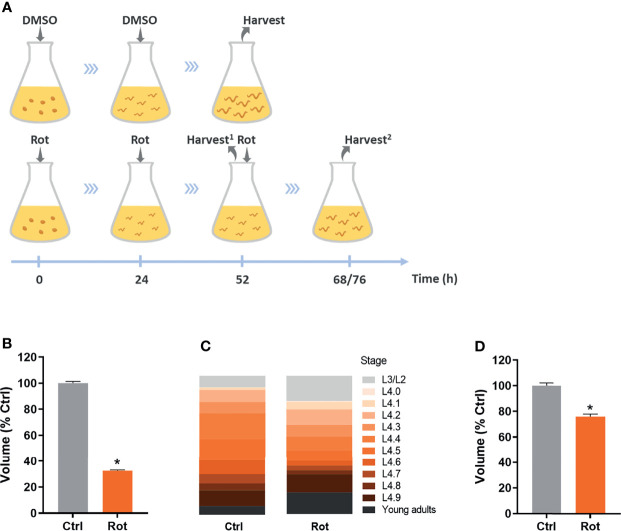
Rotenone impairs *C. elegans* growth and development. Solvent control (Ctrl) and rotenone-exposed (Rot) nematodes were assessed for size and developmental stage as described in the methods. **(A)** Representative image of the exposure design: dimethyl sulfoxide (DMSO) and 0.5 µM rotenone solutions containing 0.125% DMSO were added at the indicated time points and nematodes were harvested when most of the animals were at the L4 larval stage (Harvest2) for all the assays, except for results shown in **Figure 1B**, where animals were harvested at the same time point as the control animals (52 h; Harvest1) (see methods for further details). **(B)** Volume of nematodes exposed to rotenone for 52 h compared to solvent control (% Ctrl). **(C)** Sub-stage distribution and **(D)** volume of Ctrl and Rot-treated animals after L4 stage synchronization. * represents significant difference (p < 0.05) compared to control.

K-agar 100 mm Petri plates containing a non-starved mixed population of nematodes and plenty of embryos were washed with K-medium (51 mM sodium chloride, 32 mM potassium chloride) a few times until no animals were observed in the plates, leaving only the embryos. These plates were then used to obtain roughly age-synchronized young gravid adults 3-4 days later, which were used to obtain intact, closely age-synchronized embryos by dissolving adults for 8-10 min in freshly prepared sodium hydroxide bleach solution (3.5 mL K-medium, 500 µL 5N NaOH, 1 mL sodium hypochlorite). Age-synchronized embryos were then added to Erlenmeyer exposure flasks containing complete K-medium (51 mM sodium chloride, 32 mM potassium chloride, 3 mM calcium chloride, 3 mM magnesium sulfate, 5 µg/mL cholesterol) and live HB101 *E. coli* at a final OD_600_ of 2.0. For all assays, embryos were added at a density of 1 embryo/100 µL, except for the RNA-seq assay in which embryos were added at a density of 1 embryo/µL (it is important to note that the increased nematode density did not significantly decrease HB101 concentration; data not shown). 1 mM rotenone frozen stocks (-80°C) were previously prepared in pure dimethyl sulfoxide (DMSO), then freshly diluted with pure DMSO to obtain 500 µM rotenone, and further diluted with complete K-medium to 100 µM rotenone in 25% DMSO. This rotenone solution was then used to add to the exposure flasks to a final concentration of 0.5 µM rotenone and 0.125% DMSO as a solvent. In rotenone groups (Rot), rotenone was added at the start of the exposure and, because of its short half-life, re-added after 24 and 48 h of exposure. In the solvent control groups (Ctrl), the solvent solution (25% DMSO diluted in complete K-medium to a final concentration of 0.125%) was added at the start of the exposure and re-added after 24 h. Exposure flasks were maintained inside a 20°C incubator under constant agitation. Nematodes were then harvested when most of the animals reached the L4 larval stage, which was after 52 h for the solvent control animals (and for the growth experiment with non-stage-synchronized rotenone-exposed animals) and was either 68 or 76 h for the L4 stage-synchronized rotenone-exposed animals (**Figure 2A**).

### Nematode Growth and Developmental Stage

N2 nematodes exposed to rotenone or DMSO were harvested after 52 h and after 68/76 h (rotenone-exposed animals only) for size measurements. After exposure, animals were rinsed with K-medium and transferred to K-agar plates without any bacteria for imaging. Once the plates were air-dried, they were then placed on a Keyence BZ-X700 microscope for nematode imaging. Images were analyzed using the WormSizer (27) plugin for Fiji/ImageJ image software (28). A subset of the animals was transferred to 96 Well Microplates, µClear^®^ (Greiner Bio-One) containing 100 µL of K-medium containing 50 mM sodium azide for immobilization, and vulval development was examined to precisely determine the larval stage and L4 sub-stages of the animals (29).

### Mitochondrial Functional Parameters

N2 nematodes exposed to rotenone or DMSO were harvested once most of the animals reached the L4 stage. Nematodes oxygen consumption rate (OCR) was measured using the Seahorse Xfe24 Extracellular Flux Analyzer (Agilent Technologies, Inc.) as described by Luz and colleagues (30), except that we used the 24-well Seahorse XF24 V7 PS Cell Culture microplates, not islet plates. Pharmacological inhibitors of the electron transport chain (ETC) were used to assess different mitochondrial functional parameters. Dicyclohexylcarbodiimide (DCCD) was used to inhibit ATP synthase and provide a measure of the amount of oxygen consumption coupled to ATP production (ATP-linked OCR). Carbonyl cyanide 4-(trifluoromethoxy) phenylhydrazone (FCCP) was used to uncouple ATP production from oxygen consumption, providing a measure of maximal OCR. By subtracting the basal OCR from the maximal OCR, we calculated the spare respiratory capacity as an indication of an organism’s ability to respond to increased energy demands. Finally, sodium azide was used to inhibit cytochrome c oxidase, providing a measure of non-mitochondrial OCR, and by subtracting the azide-inhibited OCR from the DCCD-inhibited OCR we calculated proton leak. Calculations were made according to Luz and colleagues (30), with the exception that all basal OCR readings, the lowest three OCR readings after DCCD injection, and the highest three OCR readings after FCCP injection were used. Moreover, the first OCR reading after any drug injection was not considered. After the OCR readings were finalized, the number of animals in each well was counted and OCR values were normalized to the average nematode volume (WormSizer) within each experiment. At least three replicate wells were used for each inhibitor within each treatment group. The experiment was repeated at least three times.

### mRNA-seq Analysis

N2 nematodes exposed to rotenone or DMSO were harvested for mRNA-seq analysis once most of the animals reached the L4 stage. Samples containing approximately 1,000 nematodes from each group were rinsed several times with K medium (51 mM sodium chloride, 32 mM potassium chloride) until most of the bacteria were cleared out, then flash-frozen and maintained at -80°C. Only one exposure flask was used per group per experiment (no technical replicates) and the entire experiment was repeated four times using independent biological replicates (N=4 per group).

Nematode samples were then used for RNA extraction using 1 ml TRIzol Reagent (Invitrogen) according to the manufacturer’s protocol with the exception that approximately 100 µL of acid-washed sand (Sigma-Aldrich), measured with a graduated 1.7 mL microcentrifuge tube, was added to each sample at the beginning of the protocol to aid homogenization. RNA was eluted in nuclease-free water and stored at -80°C until further use. A nanodrop spectrophotometer (ThermoFisher) was used to assess the purity of the extracted RNA and the Qubit RNA HS Assay kit (ThermoFisher) was used to determine concentration. All RNA samples used for mRNA-Seq library preparation had a 260/280 absorbance ratio over 1.8. Libraries were prepared using the NEBNext Ultra II RNA Library Prep Kit for Illumina (New England Biolabs) starting with 500 ng total RNA per library and 9 cycles of PCR. Libraries were sequenced on Illumina NovaSeq 6000 to obtain 50 bp paired-end reads.

For mRNA-seq analysis, version WS273 of the *C. elegans* genome was used for mapping. Paired-end reads were mapped with bowtie (31) with the following settings: bowtie -I 0 -X 500 –chunkmbs 256 -k 1 -m 2 -S -p 2. The average mapping efficiency across all samples was 63.3% with a standard deviation of 0.74%. HTSeq version 0.11.2 (32) was used to count reads mapping to the WS273 canonical geneset. Count data was restricted to include only protein-coding genes (20,127), and then further restricted to include only genes with CPM > 1 in at least 4 libraries (14,426 genes). Differential expression analysis was then performed using edgeR version 3.24.3 (33). The web-based WormCat tool (34) was used to perform gene set enrichment analysis of the differentially expressed genes (DEG). For this analysis, we used the complete list of significantly DEG (FDR < 0.05) upon rotenone treatment which was mapped to the *C. elegans* “Whole genome v2”, following the standard WormCat Flow analysis parameters. The WormExp v 1.0 application for gene set enrichment analysis for *C. elegans* (https://wormexp.zoologie.uni-kiel.de/wormexp/) (35) and the “module-weighted annotations” analysis tool DEXICA (http://genemodules.org/) (36) were also used to further explore genes and pathways potentially involved in the rotenone-induced effects. For the WormExp analysis, only DEG annotated as immune or pathogen defense-related were used (19 genes) and query was run against the “microbes” category using the application’s default parameters. For the “module-weighted annotations” analysis, the whole transcriptomic data from solvent control *vs*. rotenone-exposed nematodes was used, following the creators’ instructions, using default parameters.

### Preparation of Pathogen Plates

*P. aeruginosa* PA14 and *S. enterica* SL1344 bacteria were picked from a frozen 25% glycerol stock, spread on a Luria-Bertani (LB) agar plate, and allowed to grow at 37°C overnight. Colony plates were stored sealed with parafilm at 4°C and used for up to one week. Bacterial cultures were prepared by placing 3–4 colonies into a 4 mL LB broth and growing at 37°C under constant shaking for 8 h. To prepare pathogen slow killing (SK) plates, modified nematode growth medium (NGM) (37) (0.35% instead of 0.25% peptone) Petri plates (35 mm) were seeded with 50 μL of each bacterial culture, which was spread over the entire plate (full lawn) using a sterile glass rod, and then placed at 37°C overnight. Freshly prepared pathogen SK plates equilibrated to room temperature were then used for the *hsp-6*p::GFP expression and survival assays.

### Survival

N2 nematodes exposed to rotenone or DMSO were harvested once most of the animals reached the L4 stage, placed into OP50 K-agar plates for 48 h, and then transferred to *P. aeruginosa* PA14 or *S. enterica* SL1344 SK plates and maintained at 25°C. Animals were transferred to fresh SK pathogen plates daily during the reproductive period. Survival was scored twice a day (early morning and late afternoon) for *P. aeruginosa* and once a day for *S. enterica*. Animals were considered dead when no pharyngeal pumping was observed, or nematodes did not respond to touch. Animals that crawled out of the agar were censored. Two or three replicate plates were used per exposure group per experiment, which was repeated three times.

### *hsp-6*p::GFP Expression

SJ4100 nematodes exposed to rotenone or DMSO were harvested once most of the animals reached the L4 stage and placed into OP50 K-agar plates for at least 1 h and then transferred to fresh *P. aeruginosa* PA14, *S. enterica* SL1344, or *E. coli* OP50 SK plates for 24 h at 25°C. At the end of the exposure, nematodes were transferred to 96 Well Microplates, µClear^®^ (Greiner Bio-One) containing 100 µL of K-medium containing 50 mM sodium azide for immobilization. Nematodes were imaged using a Keyence BZ-X700 microscope equipped with a GFP filter. Images were transformed to 32-bit and nematodes were manually outlined using the polygon selection tool using Fiji/ImageJ image software (28). Mean gray values within the selection were obtained from each nematode and subtracted from the average mean gray value from three selections of the image background. Results are shown as the fold-change of the *E. coli* OP50 solvent control group and represent 22-45 nematodes analyzed per experiment, which was repeated twice.

### Statistical Analyses

Statistical analyses were performed using GraphPad Prism 9 (GraphPad Software, LLC^©^). The size and mitochondrial functional parameters data were compared between groups using a Student’s T-test. Nematode survival was plotted as a nonlinear regression curve and the significance of differences (*p*<0.05) between survival curves were analyzed using the Mantel-Cox log-rank test. Survival scoring results of each experiment and statistical analysis of rotenone *vs*. solvent control (DMSO) animals are provided in the Supporting Information file. The time points at which mortality reaches 50% of the animals (LT50) were obtained from the best-fit IC50 value given for each curve. All curves presented R squared ≥ 0.86. The *hsp-6*p::GFP expression data were analyzed *via* two-way ANOVA followed by pairwise comparisons using Fisher’s LSD test to determine significant differences between groups (two independent variables: toxicant exposure and bacterial food source), adjusting the significance level to α = 0.05/8 = 0.00625 (Bonferroni correction) to control for multiple comparisons.

## Results

### Rotenone Impairs Growth and Developmental Rate

Age-synchronized *C. elegans* embryos were exposed to rotenone or DMSO throughout larval development (**Figure 2A**). As a first approach to assess if our rotenone developmental exposure protocol was promoting mitochondrial dysfunction, we performed nematode growth and development measurements, which are parameters commonly disturbed by mitochondrial inhibitors. As expected, nematodes exposed to rotenone presented a significantly smaller body size than animals exposed to DMSO only (**Figure 2B**). Nematode anatomic inspections using a stereo microscope allowed us to identify that rotenone-exposed animals were not only smaller but developmentally delayed (data not shown). By staging the vulval development of nematodes, we were able to determine that rotenone-exposed nematodes required on average 20 more hours to reach the mid-L4 larval stage and displayed a higher spread of animals at different stages than the solvent control group (**Figure 2C**). Nonetheless, even when rotenone-exposed nematodes were harvested at the time-point when the majority of the animals were at the L4 stage (stage-synchronized rotenone-exposed animals), they were still significantly smaller than solvent control animals (**Figure 2D**). These results suggest a rotenone-dependent impairment of mitochondrially derived energy to promote *C. elegans* growth and development.

### Rotenone Disrupts Mitochondrial Bioenergetics

**Figure 3:**
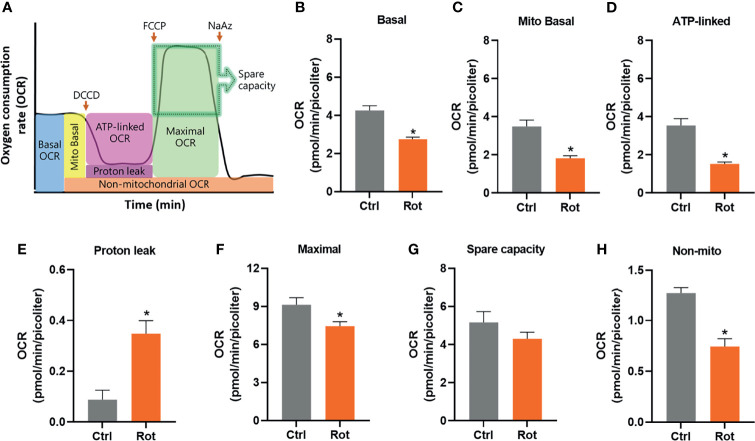
Rotenone disrupts *C. elegans* mitochondrial function. Solvent control (Ctrl) and rotenone-exposed (Rot) nematodes were assessed for oxygen consumption using a Seahorse XFe24 bioanalyzer as described in the methods. Oxygen consumption rates (OCR) were normalized to the average nematode volume within each experiment. **(A)** Representative image of the mitochondrial function parameters assessed using the inhibitors dicyclohexylcarbodiimide (DCCD), carbonyl cyanide 4-(trifluoromethoxy) phenylhydrazone (FCCP), and sodium azide (NaAz). **(B)** Total basal OCR, **(C)** mitochondrial basal OCR, **(D)** ATP-linked OCR, **(E)** proton leak, **(F)** maximal OCR, **(G)** spare respiratory capacity, and **(H)** non-mitochondrial OCR are presented. Student’s T-test was performed for each parameter. Bars represent mean ± SEM. * represent significant difference (p < 0.05) between groups.

Real-time oxygen consumption of live nematodes was monitored in rotenone or DMSO-exposed animals at the L4 larval stage using different mitochondrial inhibitors (**Figure 3A**). Total basal, mitochondrial basal, and ATP-linked OCR were all significantly lower in rotenone-exposed animals (**Figures 3B–D**). Rotenone also caused an increase in proton leak (**Figure 3E**), and a decrease in maximal OCR (**Figure 3F**), while no significant changes were detected in the spare respiratory capacity (**Figure 3G**). The non-mitochondrial OCR was also significantly reduced in rotenone-exposed animals (**Figure 3H**). Together, these results reveal a rotenone-induced disruption of *C. elegans* mitochondrial bioenergetics towards a lower mitochondrial efficiency (mitochondrial respiration coupled to ATP production), which are in line with the abovementioned findings on growth impairment.

### Rotenone Alters the Transcript Levels of Genes Related to Metabolism, Immune and Stress Response Pathways

**Figure 4:**
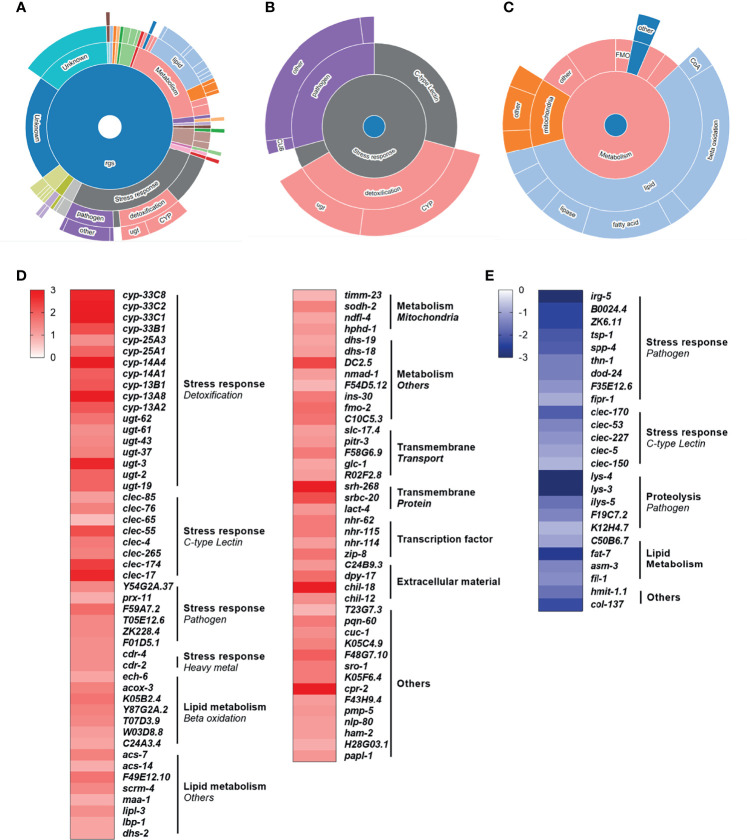
Rotenone modulates genes related to metabolism, immune and stress response pathways. All 179 differentially expressed genes (DEG) from the rotenone treatment were submitted to WormCat analysis for annotation and visualization of enrichment data. **(A)** Sunburst diagram showing significantly enriched categories, highlighting the two major broad enriched categories: **(B)** stress response and **(C)** metabolism. Heatmap representing the log fold-change of **(D)** up-regulated and **(E)** down-regulated genes with known function are shown grouped by gene categories (bold) and subcategories (italic).

To investigate the role of specific mitochondrial disruption on modulating conserved immunometabolic molecular pathways, we performed a whole transcriptome mRNA-seq analysis. Our results revealed that rotenone-treated animals displayed a total of 179 differentially expressed genes (DEG), among which 134 were up-regulated (**Supplementary Table 1**) and 45 were down-regulated (**Supplementary Table 2**). By applying the WormCat gene enrichment analysis tool, we detected an enrichment of genes involved in detoxification, energy metabolism, or pathogen defense (**Figures 4A–C**). Among the known genes which were up-regulated, we can highlight genes involved in detoxification, from the cytochrome P450 (CYP) and UDP-glucuronosyltransferase (UGT) families, as well as beta-oxidation and other lipid and mitochondrial metabolism pathways (**Figure 4D**). Despite a few genes associated with pathogen defense being represented within the up-regulated list of genes, interestingly, these comprised the majority of the down-regulated set of genes (**Figure 4E**). Some genes involved in lipid metabolism were also down-regulated, with *fat-7* being found as the most largely down-regulated gene (**Figure 4E**). This gene-category enrichment analysis, thus, revealed a major role of rotenone in modulating the expression of several genes related to immune and metabolic function.

We further applied our whole transcriptome data (log fold-change of all 14,426 quantified genes) to the “module-weighted annotations” analysis tool 1 (36). This analysis compares transcriptomic data to 209 modules that represent patterns of transcriptional regulation (co-expressed genes) in *C. elegans*, representing different conditions such as responses to changes in the environment (*e.g.*, starvation, exposure to xenobiotics), genes regulated by transcriptions factors (*e.g.*, ATFS-1, DAF-16), genes specific to tissues (*e.g.*, neurons, muscle), genes that change during development, and other complex transcriptional responses to genetic, environmental and temporal perturbations. Results from our data set revealed four significantly active modules (**Supplementary Figure 1A**), representing: (i) activation of the UPR^mt^, evidenced by a significantly positive association with module #47; (ii) activation of neuronal genes and neuronal peptide signaling, evidenced by a significantly positive association with module #23; and (iii) suppression of hypoxia-inducible factor (HIF-1)-dependent genes, evidenced by a significantly negative association with modules #66 and #169. A list with the main gene ontology process, molecular function, and cellular component associated with each active module is provided in **Supplementary Figure 1B**. Thus, this analysis allowed us to identify two conserved immunometabolic pathways, the UPR^mt^ and HIF-1 regulatory pathways, that are potentially being disturbed upon rotenone-induced mitochondrial dysfunction.

### Rotenone Alters Nematode Susceptibility to Pathogens

**Figure 5:**
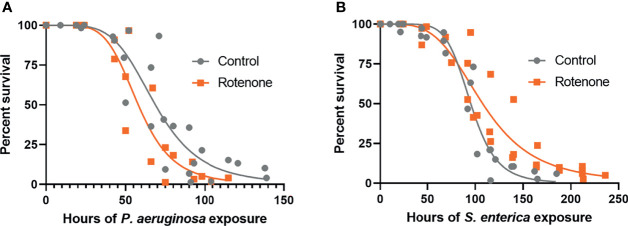
Rotenone alters *C. elegans* pathogen susceptibility. Solvent control (Control) and rotenone-exposed nematodes (Rotenone) were further continuously exposed to **(A)** *Pseudomonas aeruginosa* (PA14) or **(B)** *Salmonella enterica* (SL1344) and scored for survival. Curves were fit by nonlinear regression based on the results from three independent experiments for each pathogen exposure and represent a total of 148-197 animals scored (already excluding censored animals) per group. Details about the number of animals in each experiment and statistical results using the Mantel-Cox log-rank test are shown in **Supplementary Table 1**.

To address if the rotenone-induced modulation of immuno-metabolic pathways observed at the gene expression level could translate to organismal-level effects on pathogen susceptibility, DMSO or rotenone-exposed animals were challenged with the gram-negative pathogens *P. aeruginosa* (PA14) or *S. enterica* (SL1344). Survival results revealed that while rotenone-exposed nematodes were more susceptible to *P. aeruginosa* (**Figure 5A**), they were overall more resistant to *S. enterica* (**Figure 5B**). Based on the curve fit data, we were able to obtain the time point at which mortality reaches 50% of the animals (LT50), which was 68.7h and 58.0h for solvent control and rotenone-exposed nematodes, respectively, challenged with *P. aeruginosa*. This corresponds to a 16% decrease in life expectancy for half of the population tested caused by the rotenone exposures. On the other hand, nematodes challenged with *S. enterica* presented a 12% increase in LT50 (95.3h and 107.8h for solvent control and rotenone-exposed nematodes, respectively). These results reveal that a specific mitochondrial dysfunction induced by rotenone can alter *C. elegans* pathogen susceptibility.

### The Gene *hsp-6* Is Upregulated by Rotenone Exposure During Pathogen Infections

**Figure 6:**
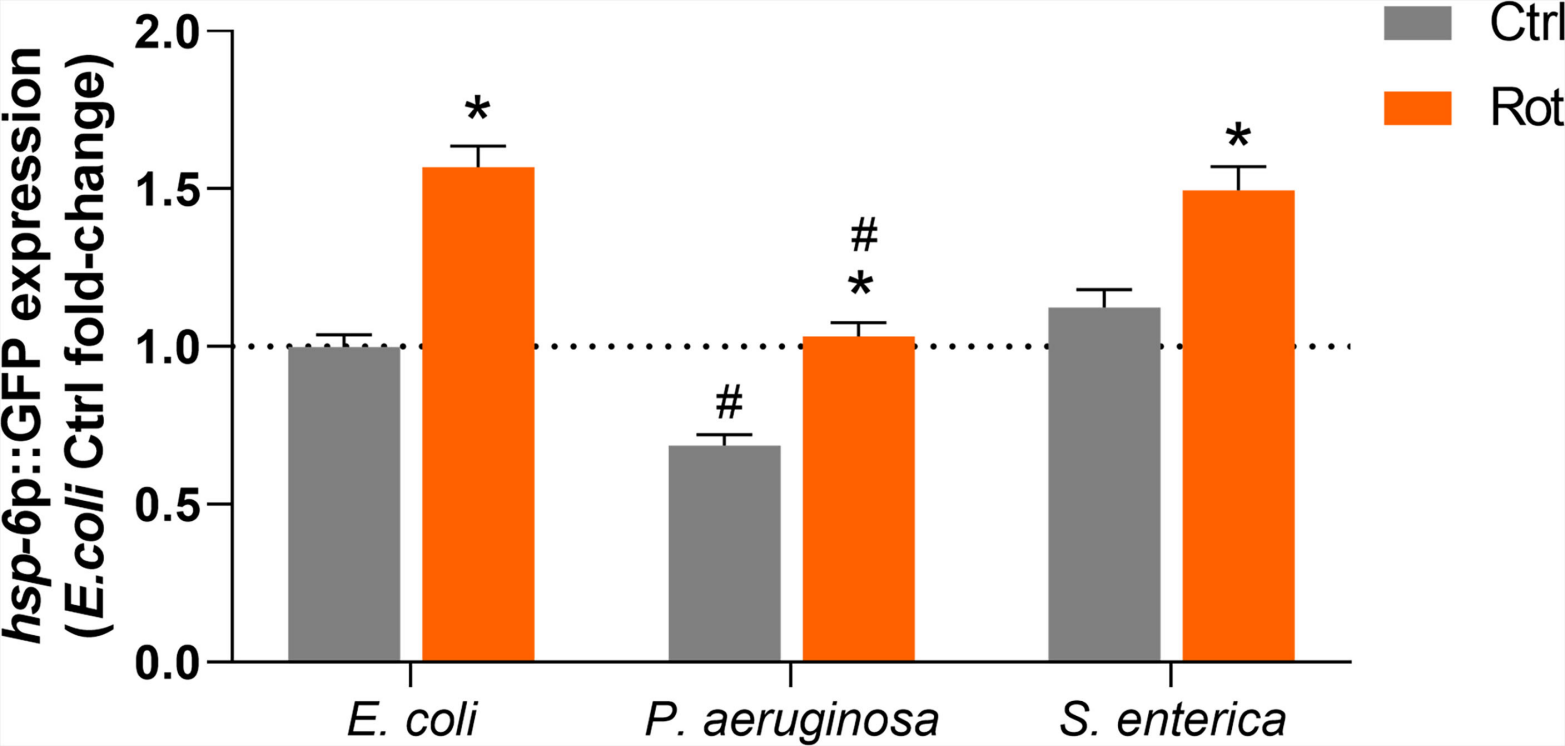
Activation of the mitochondrial unfolded protein response by rotenone. Solvent control (Ctrl) and rotenone-exposed (Rot) SJ4100 nematodes were further exposed for 24 h to *Escherichia coli* (OP50), *Pseudomonas aeruginosa* (PA14) or *Salmonella enterica* (SL1344) and the GFP intensity was measured. The presented *hsp-6*p::GFP fold-change results are relative to the *E. coli* OP50 solvent control. A two-way ANOVA with Bonferroni multiple comparison test was performed. Bars represent mean ± SEM. * represent significant difference (p < 0.05) between the rotenone and solvent control exposure groups within each bacterial feeding group. # represent significant difference (p < 0.05) comparing pathogenic bacterial exposures to *E. coli* within the solvent control or rotenone exposure groups. No significant interaction effect between the two independent variables – ”toxicant exposure” and “bacterial food source” – was detected (*p* = 0.09). However, each independent variable had a significant effect on *hsp-6*p::GFP expression (*p* < 0.0001 for both independent variables).

The potential role of the mitochondrial unfolded protein response (UPR^mt^) pathway in the differential pathogen susceptibility of rotenone-exposed animals to *P. aeruginosa* or *S. enterica* was investigated by quantification of *hsp-6*p::GFP expression in DMSO and rotenone-treated nematodes further exposed for 24 h to *E. coli* (OP50), *P. aeruginosa* (PA14) and *S. enterica* (SL1344). *hsp-6*p::GFP expression was significantly upregulated in rotenone-treated animals at similar levels (1.4-1.5 fold-induction) in all bacterial exposures (**Figure 6**). Nonetheless, *P. aeruginosa* exposure alone (solvent control animals) suppressed *hsp-6*p::GFP expression in comparison to animals that were fed the control bacteria *E. coli* (OP50), and rotenone treatment was able to restore *hsp-6*p::GFP expression only to the level of solvent control OP50-fed nematodes (**Figure 6**). In line with the “module-weighted annotations” analysis, these results are also consistent with an activation of the UPR^mt^ pathway by rotenone. Nonetheless, this apparent activation is limited in the presence of *P. aeruginosa*, but not *S. enterica*, which could partly explain our findings on differential pathogen susceptibility.

## Discussion

Previous studies from our research group and data from the literature show that different classes of toxicants can promote physiological stress in organisms by changing energy metabolism (14, 38–41) and innate immune responses (1–7, 42–48). However, although the fields of immunometabolism and mitoimmunity have recently been the focus of extensive research, most studies use vertebrate models, and little attention is directed to mitoimmune dysfunction in the context of pollutant exposures.

To start tackling the hypothesis that mitochondrial toxicants can also act as immunometabolic disruptors and alter disease susceptibility, we exposed nematodes to the pesticide rotenone, which is a classic and specific mitochondrial complex I inhibitor. In this study, we opted to perform a developmental rotenone exposure because, at this stage, animals are usually more sensitive to mitochondrial toxicants. This greater sensitivity is because while early developmental stages and growth involve biological processes which require high levels of mitochondrially-derived energy, at these stages, several mitochondrial parameters are limited or greatly reduced (49–53). Moreover, mitochondrial parameters and function are highly variable with developmental stage; thus, it is likely that mitochondrial toxicants can have more profound effects on some developmental windows of susceptibility (14, 41). Furthermore, it is expected and frequently observed that defects should occur during exposures at these early life stages with mitochondrial toxicants (40, 54). Indeed, our results demonstrated that rotenone caused significant impairment in growth (**Figures 2B–D**) and bioenergetic efficiency (**Figures 3A–E**). Associations between growth and decreases in mitochondrial efficiency (respiration coupled to ATP production) have already been reported (55, 56). It is important to highlight, however, that despite these rotenone-induced effects in growth and mitochondrial function, no mortality was observed, and animals were able to fully develop to adulthood and reproductive maturation without affecting total brood size (pilot study data in **Supplementary Figure 2**). Sublethal rotenone exposures as well as mild inhibition of mitochondrial complex I genes have been reported to increase *C. elegans* lifespan (57–59). Therefore, it is reasonable to believe that, with our rotenone exposure protocol, we did not cause a severe and generalized organismal toxicity but rather a relatively mild mitochondrial stress.

Next, we investigated if this mild and specific mitochondrial stress in rotenone-exposed nematodes could lead to the modulation of immunometabolic pathways through transcriptomic analysis. Gene ontology analysis of the DEG revealed that the great majority of the known genes were implicated in energy metabolism and stress response (**Figure 4A**). Most of the DEG associated with energy metabolism were up-regulated and annotated as involved in fatty acid β-oxidation (**Figure 4C**). This result is consistent with a previous study from our group which also detected, through metabolomics analysis, increased fatty acid β-oxidation in adult nematodes exposed to rotenone (39). This increase might be related to a need to replenish acetyl-CoA levels or to boost ATP production upon Complex I inhibition, although the latter hypothesis was not confirmed in our previous work (39). Fatty acid β-oxidation plays a significant role in immunometabolism. For example, increased fatty acid β-oxidation is important for innate immune responses in higher vertebrates such as macrophage polarization towards an anti-inflammatory state (60). In *C. elegans*, although, to our knowledge, direct associations between fatty acid β-oxidation and innate immunity have not been yet explored, several studies describe the importance of certain fatty acids for pathogen resistance [see review (17)]. For example, the fatty acid oleate, which is synthesized by *fat-6* and *fat-7* desaturases, is required in *C. elegans* for the pathogen-mediated induction of immune defense genes, and disruption of oleate synthesis increase nematode susceptibility to *P. aeruginosa* (61). The *fat-7* gene was highly down-regulated by our rotenone treatment, which could have contributed to our observations of increased susceptibility of the rotenone-exposed nematodes to this same pathogen (**Figure 5A**). Additionally, *fat-7* down-regulation was reported to cause growth delay, reduced size, and increased expression of genes in β-oxidation (62, 63). Interestingly, all of these phenotypes were also observed in our rotenone-treated nematodes. These literature reports, together with our findings, indicate that *fat-7* down-regulation –thus potentially oleate synthesis disruption– might be a central player governing the rotenone-induced effects on immunometabolic and pathogen susceptibility responses in *C. elegans*.

The stress-response DEGs were further separated into three subcategories: detoxification, C-type lectins, and pathogens (**Figure 4B**). Among the xenobiotic metabolism (“detoxification”)-related DEG are genes belonging to the Cytochrome P450 (CYP) and UDP-glucuronosyltransferase (UGT) families, which were identified as up-regulated by our rotenone treatment (**Figure 4D**). The enzymatic modification (biotransformation) of rotenone in rats and fish has already been shown to involve oxidation by CYPs (64). Evidence of rotenone detoxification by CYPs was also reported in invertebrates such as insects and helminths (65, 66). While we could not find studies describing the direct involvement of UGTs in rotenone detoxification, similarly to our findings, rotenone also induced several UGT genes in the fruit fly *Drosophila melanogaster* (67). These results suggest that the mechanisms of rotenone biotransformation might be conserved amongst vertebrates and invertebrates, although in general the *C. elegans* xenobiotic biotransformation processes, and in particular the substrate specificities of the relevant enzymes, are relatively poorly understood (68). Together with our growth and mitochondrial dysfunction observations, these findings strengthen the suitability of the *C. elegans* model to address conserved modes of action of mitotoxicants.

It is not surprising that exposure to a xenobiotic that inhibits mitochondrial function elicited a transcriptomic response fitting with metabolic restructuring and xenobiotic metabolism/detoxification. Perhaps more surprisingly, we also saw gene expression changes consistent with altered immune responses. For example, among the other prominent stress-response-related subcategories were “C-type lectins”. These pattern recognition receptors (PRRs) are well-recognized for their role in the innate immune response. They can detect pathogen-derived carbohydrate ligands and activate immune signaling pathways to protect against infection (69, 70). In *C. elegans*, to our knowledge, the specific function of proteins belonging to this family has not been characterized. Nonetheless, it has been suggested based on sequence analogy, that several *C. elegans* C-type lectins may have a role in recognizing non-self carbohydrates (71). Most of the C-type lectin DEG in our database were up-regulated (**Figure 4D**), but a few of them were also found to be down-regulated (**Figure 4E**). It would be interesting to investigate if some of these differentially-expressed C-type lectins could be specific to one or the other pathogen used in this study and potentially involved in the rotenone-induced differential pathogen resistance phenotype. Although, to our knowledge, the role of C-type lectins in immunometabolism has not been much explored, there is evidence of metabolic changes of vertebrate immune cells induced through C-type lectin-signaling pathways (72). Thus, future studies should further explore the role of this PRR family in the metabolic regulation of *C. elegans*.

The final of the three most represented stress-response-related subcategories was “Pathogens”. This subcategory comprised only a few genes that were up-regulated (**Figure 4D**) with a majority that were down-regulated (**Figure 4E**), suggesting a role of rotenone in shifting nematodes to a more immunosuppressive state. To compare how this set of genes is usually modulated during infections, we took advantage of another *C. elegans* bioinformatics tool, WormExp (35), and performed a gene enrichment analysis using a list comprising only our DEG related to innate immunity and/or pathogen defense (19 genes which are highlighted in **Supplementary Tables 1, 2** and from now on described as “immune-related DEG”). We have identified significant overlap with different gene expression data sets related to *P. aeruginosa* infection and, interestingly, all of these data sets were described as up-regulated upon *P. aeruginosa* infection (**Supplementary Table 2**). By compiling all our immune-related DEG that overlapped with these data sets describing genes up-regulated upon *P. aeruginosa* infection, we obtained a list of fifteen genes in total and found that eleven of these were downregulated upon rotenone treatment. In fact, two of them were among the list of genes confirmed for *P. aeruginosa* resistance: *dod-24* and *irg-5* (73). This major role of rotenone in suppressing immune-related pathways and specific genes up-regulated by *P. aeruginosa* might contribute to the observed increased susceptibility to *P. aeruginosa* infection (**Figure 5A**). In another study, however, Chikka et al. (74) found that rotenone exposures induced a distinct immunomodulatory response in *C. elegans*. Rotenone activated the p38-MAPK pathway, which is a conserved immune-related pathway required for resistance to a diverse range of pathogens (75). Interestingly, none of the p38-MAPK-dependent genes that were up-regulated upon their rotenone exposures were differentially-expressed within our dataset. However, it is unknown whether this rotenone-induced p38-MAPK pathway activation altered the ability of nematodes to survive *P. aeruginosa* infections. Similarly to the findings from Chikka et al. (74), Campos et al. (59) reported that a point mutation in the complex I subunit gene *nuo-6* which resulted in a mild impairment of mitochondrial function also activated the p38-MAPK pathway. Moreover, these mutants were more resistant to *P. aeruginosa* infections (59). Although the results from these two studies may seem to contradict our findings, it is important to note that the exposure conditions from Chikka et al. (74) were very distinct from our study including differences in rotenone concentration, developmental exposure window, and the nematode age at which these gene expressions were assessed. Similarly, in the study from Campos et al. (59), *nuo-6* mutants were subjected to a continuous mitochondrial impairment throughout their lifetime and for several generations, thus probably eliciting a series of adaptive molecular mechanisms, which may not be present during a time-limited chemically-induced mitochondrial impairment. Most importantly, however, all these studies are in line with a key role of mitochondrial dysfunction in the modulation of immune pathways in the *C. elegans* model.

Despite the down-regulation of several immune-related genes induced by rotenone, exposed nematodes did not display increased susceptibility to *S. enterica*. In fact, in two out of our three experiments, nematodes displayed a clear and significant increased resistance (**Figure 5B** and **Supplementary Table 4**). Unfortunately, we were unable to gain any mechanistic insights using the WormExp tool, as we did not identify any significant overlap with gene data sets related to *S. enterica* infection. This is probably because, to our knowledge, to this date, the WormExp database lacks gene sets with this pathogen species alone (there are some data sets available, but they represent combined treatments, *e.g.*, “UP by *S. enterica* SL2048 under *fer-1* mutant”).

In *C. elegans*, *S. enterica* and *P. aeruginosa* trigger distinct pathogen-defense mechanisms. For example, the ZIP-2 transcription factor is required for resistance against PA14 and regulates the expression of *irg-1*. *irg-1* is up-regulated by PA14, but not *S. enterica* and other less pathogenic strains of *P. aeruginosa* (76). Moreover, in *C. elegans*, the pathogenicity of *S. enterica* relies on disseminated oxidative stress during infection (77), whereas such prominent oxidative stress levels, to our knowledge, have not been described during *P. aeruginosa* infections. With such distinct mechanisms of pathogenicity, it is reasonable to expect that host survival will also be dependent on different defense-related pathways.

One mechanism evolved in *C. elegans* to survive pathogens is an evasion strategy, also known as avoidance behavior, which is chiefly mediated by chemosensory neurons (78). Pathogen avoidance plays a significant role in the *C. elegans* defense response to *P. aeruginosa* but not to *S. enterica* (79). Therefore, one could argue that the differential pathogen susceptibility response caused by rotenone in this study could be related to effects on avoidance behavior. Indeed, chemicals may impact chemosensory behavior controlling the *C. elegans* ability to be attracted or avoid certain natural products. For example, we have recently shown that a sublethal dose of silver nanoparticles can completely block the *C. elegans* ability to be attracted to a food-like odor (80). Contributing to this idea is the fact that rotenone is also well known for causing neurotoxic effects (81). Having this in mind, all our pathogen challenges were conducted in Petri plates seeded with each bacterial culture spread over the entire plate (full lawn). With this approach, potential effects on pathogen avoidance are not likely to have affected our survival results.

Colonization and proliferation of bacterial pathogens in the *C. elegans* intestine causes luminal distension and correlates with *C. elegans* death (82). For example, *S. enterica* typically causes a remarkable luminal distention in nematodes, and *S. enterica* mutants that are less lethal to nematodes exhibit reduced colonization capabilities (82). Thus, it would be interesting to address in future studies if the increased resistance of rotenone-exposed animals to *S. enterica* could be related to an enhanced ability of the rotenone-exposed worms to destroy and eliminate bacterial cells and prevent gut colonization. Similarly, it would be interesting to test whether the increased susceptibility to *P. aeruginosa* could be related to a decreased capability of *C. elegans* exposed to rotenone in controlling intestinal bacterial colonization and proliferation.

Our results suggest that the UPR^mt^ pathway could be involved in our rotenone-induced differential pathogen susceptibility. This pathway is probably the most studied in *C. elegans* linking mitochondria and innate immunity. Upon mitochondrial stress, the ATFS-1 transcription factor is translocated to the nucleus and triggers the expression of mitochondrial protective and immune-related genes (22, 59, 83). In our study, this pathway was shown to be up-regulated by rotenone which was evidenced in one of our transcriptomic dataset analyses (“module-weighted annotations” analysis; **Supplementary Figure 1**). This induction was sustained even after 24 h of *S. enterica* exposure. However, the UPR^mt^ was suppressed by *P. aeruginosa*, and rotenone was only able to restore it to the same levels of solvent control animals (not exposed to rotenone) (**Figure 6**). Indeed, the role of *P. aeruginosa* in suppressing the *C. elegans* UPR^mt^ was already reported (84). Therefore, it is possible that the rotenone-induced activation of UPR^mt^ was involved in the higher resistance of nematodes to *S. enterica* but was not sufficiently up-regulated during the *P. aeruginosa* infection to promote protection. Moreover, *P. aeruginosa* is known to produce mitotoxic compounds (85, 86) and cause mitochondrial dysfunction (84, 87, 88), which suggests that rotenone-exposed nematodes further infected with *P. aeruginosa* might be suffering from a double-hit in mitochondria, possibly contributing to high energy deficits and death. Corroborating to this hypothesis, Revtovich et al. (87) showed that *C. elegans* fed with an *E. coli* strain that disrupts mitochondrial homeostasis are more susceptible to *P. aeruginosa*. Nonetheless, future studies are needed to address the double-hit hypothesis between mitotoxicant chemical exposures and pathogens that target mitochondrial function and its role in promoting premature death.

Pesticides are one of the most common and important classes of pollutants in the environment, mostly due to their intensive use in agriculture worldwide (89). Although these substances have societal benefits, their use and limitations in describing their negative effects pose serious risks to human, animal, and environmental health. Importantly, besides pesticides, several other classes of environmental pollutants are also known to cause mitochondrial toxicity (14, 15). Here, we provide evidence that mitotoxicant compounds have the potential to act as immunometabolic disruptors and affect host-pathogen interactions altering disease susceptibility, highlighting the importance of careful regulation of the production and use of mitotoxic chemicals. This work also opens new roads for future studies to further characterize *C. elegans* immunometabolic pathways, which are currently understudied. It also provides potential pathways implicated in pathogen resistance and susceptibility, which can be further investigated, shedding light on potential therapeutic targets.

## Data Availability Statement

The original contributions presented in the study are publicly available. This data can be found here: GEO database; accessions GSE195584; GSM5841177-GSM5841184.

## Author Contributions

DM designed the experiment, performed the experiments and data analysis, prepared the graphical art, and wrote the main manuscript. CB contributed with experimental design and with performing experiments and data analysis. KF, RC, SB, YW, and AC contributed with experiments and data analysis. LB and JM contributed with experimental design, reagents, materials, and analysis tools. All authors contributed to the article and approved the submitted version.

## Funding

This work was funded by the National Institute of Health (R01ES028218 and P42ES010356). Some strains were provided by the Caenorhabditis Genetics Center, which is funded by NIH Office of Research Infrastructure Programs (P40 OD010440).

## Conflict of Interest

The authors declare that the research was conducted in the absence of any commercial or financial relationships that could be construed as a potential conflict of interest.

## Publisher’s Note

All claims expressed in this article are solely those of the authors and do not necessarily represent those of their affiliated organizations, or those of the publisher, the editors and the reviewers. Any product that may be evaluated in this article, or claim that may be made by its manufacturer, is not guaranteed or endorsed by the publisher.
