# Supplementary material for: Rotenone Modulates Caenorhabditis elegans Immunometabolism and Pathogen Susceptibility
Source: Front Immunol. 2022 Feb 22;13:840272. doi: 10.3389/fimmu.2022.840272 (PMC8902048; doi:10.3389/fimmu.2022.840272)
Supplement: Supplementary file 4 [file DataSheet_4.docx]

Supplementary Material

#
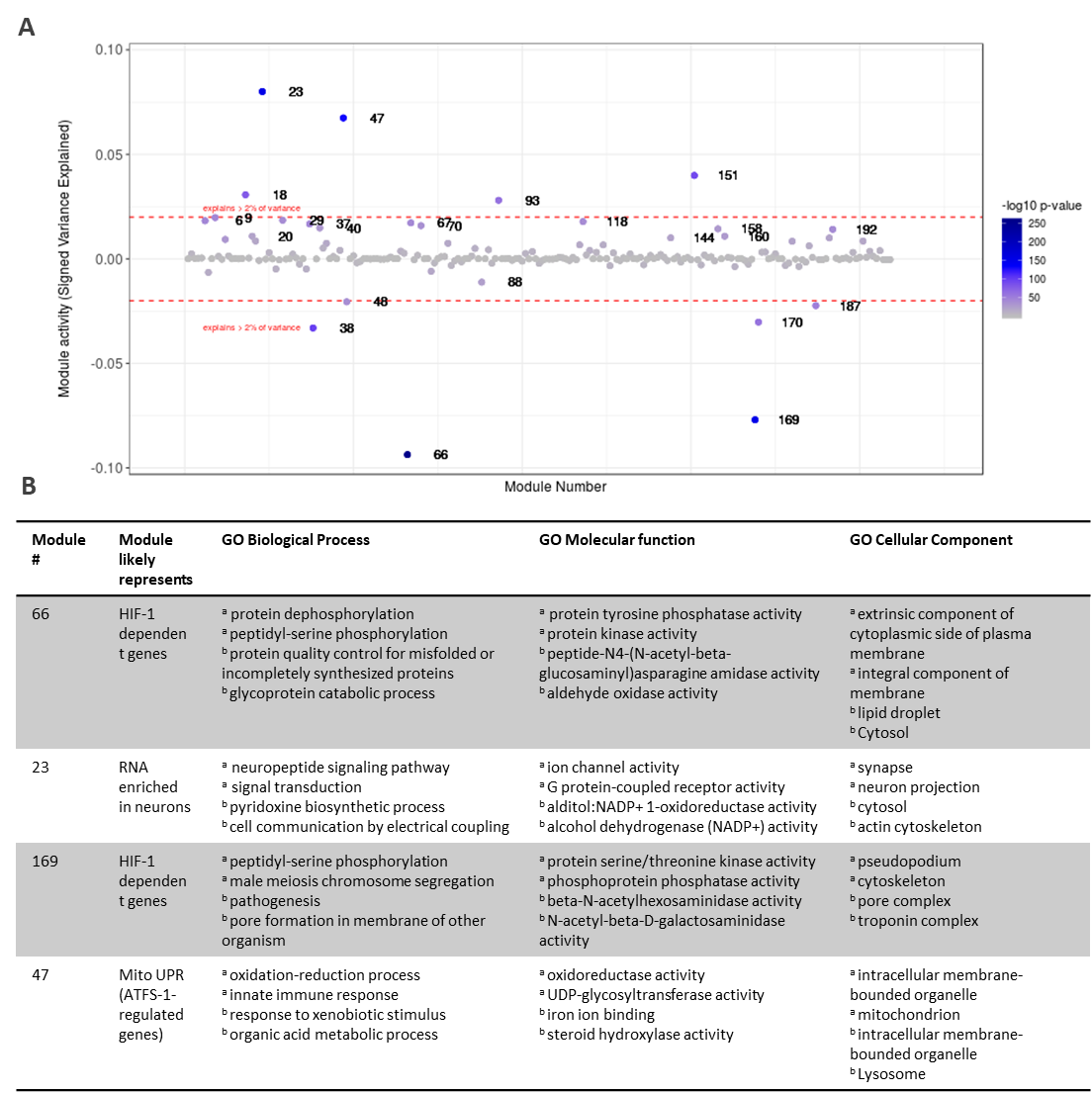
Supplementary Figures

**Supplementary Figure 1.** ***C. elegans* active modules induced by rotenone.** The whole transcriptomic data from control vs. rotenone-exposed nematodes was submitted to the C. elegans gene-modules analysis tool DEXICA (http://genemodules.org/). (a) Active modules in our gene expression data are provided as a module activity plot which is expressed as signed variance explained (SVE). (b) Modules that explains at least 5 % of gene expression variance (i.e., SVE > 0.05) are listed together with the biological phenomenon that the module most likely represents, and the top 2 GO terms enriched in each module and its respective hemi-module (a and b).

**Supplementary Figure 2.** ***C. elegans* brood size after rotenone exposures.** Nematodes exposed to the solvent control (0) or different rotenone concentrations (0.03-1 µM) were added individually to 6 cm Petri plates containing *E. coli* OP50 and transferred daily to new plates until reproduction ceased. Plates containing offspring were stored at 20 °C for two days, and then counted to obtain the total progeny number. Results (average progeny number ± standard error of the mean) are from one experiment performed with nine technical replicates per group (nine Petri plates with one nematode each). A one-way ANOVA with Dunnett’s multiple comparison test was performed and no significant differences were observed between the groups.

# Supplementary Tables

# Supplementary Table 1. Pathogen survival results and statistical analysis of rotenone *vs*. control animals. Solvent control (Ctrl) and rotenone-exposed (Rot) nematodes were further continuously exposed to *Pseudomonas aeruginosa* (PA14) and *Salmonella enterica* (SL1344) and scored for survival. Curves were compared using the Mantel-Cox log-rank test. The total number (n) of scored and censored (in parenthesis) animals, the median and maximal survival in hours, and the *p* value are provided for each experiment.

| **Pathogen** | **Expt#** | **Ctrl (n)** | **Rot (n)** | **Ctrl (median)** | **Rot (median)** | **Ctrl (maximal)** | **Rot (maximal)** | **p value** |
| --- | --- | --- | --- | --- | --- | --- | --- | --- |
| *P. aeruginosa* | 1 | 47(13) | 53(7) | 80 h | 71 h | 90 h | 66 h | 0.005 |
|  | 2 | 68(6) | 73(4) | 50 h | 43 h | 138 h | 115 h | 0.0006 |
|  | 3 | 73(25) | 71(28) | 74 h | 67 h | 104 h | 104 h | 0.02 |
| *S. enterica* | 1 | 37(1) | 37(1) | 95 h | 140 h | 165 h | 212 h | <0.0001 |
|  | 2 | 57(10) | 73(26) | 98 h | 98 h | 185 h | 188 h | 0.5870 |
|  | 3 | 54(6) | 55(5) | 92 h | 92 h | 102 h | 236 h | <0.0001 |

**Supplementary Table 2. WormExp gene enrichment results.** A list of 19 immune-related differentially expressed genes (highlighted in Supplementary Excel File 1) were submitted to gene enrichment analysis using the WormExp tool (https://wormexp.zoologie.uni-kiel.de/wormexp/). The table shows results of gene expression data sets that presented significant overlap (Bonferroni-corrected p values and FDR values <0.05) with our list of genes and matched the terms *P. aeruginosa* or PA14.

| **Category** | **Term** | **Counts** | **ListSize** | **PopHit** | **Pop Size** | **Pvalue** | **Bonferroni** | **FDR** | **Refs** |
| --- | --- | --- | --- | --- | --- | --- | --- | --- | --- |
| Microbes | UP by PA14, 12h | 13 | 33 | 285 | 29469 | 1.57E-16 | 3.60E-13 | 7.21E-14 | http://www.ncbi.nlm.nih.gov/pubmed/27066825 |
| Microbes | UP by PA14, 24h | 13 | 33 | 534 | 29469 | 2.78E-13 | 6.38E-10 | 5.80E-11 | http://www.ncbi.nlm.nih.gov/pubmed/27066825 |
| Microbes | Responding to *P. aeruginosa* | 10 | 33 | 354 | 29469 | 1.41E-10 | 3.23E-07 | 7.69E-09 | http://www.ncbi.nlm.nih.gov/pubmed/22514739 |
| Microbes | UP by PA14 (Miller) | 9 | 33 | 236 | 29469 | 1.76E-10 | 4.02E-07 | 8.70E-09 | http://www.ncbi.nlm.nih.gov/pubmed/26360906 |
| Microbes | PA14 Infection induced | 7 | 33 | 195 | 29469 | 7.42E-08 | 1.70E-04 | 1.75E-06 | http://www.ncbi.nlm.nih.gov/pubmed/16968778 |
| Microbes | UP PMK-1 and confirmed for resistance to PA14 | 5 | 33 | 38 | 29469 | 9.36E-08 | 2.15E-04 | 2.16E-06 | http://www.ncbi.nlm.nih.gov/pubmed/17096597 |
| Microbes | UP to PA14 | 7 | 33 | 258 | 29469 | 3.86E-07 | 8.85E-04 | 7.66E-06 | http://www.ncbi.nlm.nih.gov/pubmed/17096597 |
| Microbes | UP in Slow Killing, *P. aeruginosa* PA14 | 6 | 33 | 157 | 29469 | 8.47E-07 | 0.001942 | 1.46E-05 | https://www.ncbi.nlm.nih.gov/pubmed/28662060 |
| Microbes | UP by *P. aeruginosa* (Bond) | 6 | 33 | 258 | 29469 | 9.61E-06 | 0.022036 | 1.27E-04 | http://www.ncbi.nlm.nih.gov/pubmed/25474640 |
| Microbes | UP by *P. aeruginosa* PA14 (Head) | 5 | 33 | 123 | 29469 | 1.08E-05 | 0.024689 | 1.39E-04 | https://www.ncbi.nlm.nih.gov/pubmed/27600703 |
